# Assessment of Functioning and the Integration of Patients After Traumatic Brain Injury in Their Homes and Social Environments Following Inpatient Rehabilitation

**DOI:** 10.3390/healthcare12222211

**Published:** 2024-11-06

**Authors:** Neža Hrastar, Klemen Grabljevec, Gaj Vidmar

**Affiliations:** 1 Department for Rehabilitation of Patients after Traumatic Brain Injury, University Rehabilitation Institute, 1000 Ljubljana, Slovenia; neza.hrastar@ir-rs.si; 2 Department of Biostatistics and Scientific Informatics, University Rehabilitation Institute, 1000 Ljubljana, Slovenia; gaj.vidmar@ir-rs.si; 3 Institute for Biostatistics and Medical Informatics, Faculty of Medicine, University of Ljubljana, 1000 Ljubljana, Slovenia; 4 Department of Psychology, Faculty of Mathematics, Natural Sciences and Information Technologies, University of Primorska, 6000 Koper, Slovenia

**Keywords:** brain injury, community integration, questionnaires, Slovenia

## Abstract

Introduction: Patients with head injuries usually return to their home environments after completing rehabilitation, which is an additional challenge in the case of functional, motor or cognitive impairment. In order to identify the problems that such patients face and to objectify their problems with integration, we used the Community Integration Questionnaire-Revised (CIQ-R). At the same time, the validity of the translation of the questionnaire into Slovenian was checked. Methods: The CIQ-R was translated, and patients were telephoned twice, 14 days apart. In addition, they were assessed using the Functional Independence Measure (FIM) during the first call. The reliability of the CIQ-R in terms of internal consistency was assessed using Cronbach’s alpha and Guttman’s lambda-2. The reliability in terms of repeated measures was assessed using intraclass correlation (ICC). The association between FIM and CIQ-R scores was assessed using Pearson’s correlation. Results: Internal consistency of the CIQ-R was high (*α* = 0.8, *λ* = 0.85), and there was a very high re-test stability of the overall CIQ-R score (ICC = 0.95). The cognitive component of the FIM in the home environment was linearly associated with the CIQ-R score (*r* = 0.8, *p* ≤ 0.001). Conclusions: The translation of the CIQ-R questionnaire into Slovenian was successfully validated, and we proved its potential suitability for clinical use.

## 1. Introduction

All patients face difficulties when integrating back into their home environments after being discharged from the hospital. This is even more difficult after a prolonged illness or if there is an impairment that affects cognitive, behavioral and functional aspects, such as a head injury. During the acute phase in the hospital, we focus on survival, and later, during the rehabilitation, we focus on setting relatively short-term goals, always keeping in mind the patient’s eventual return to their home environment and facing the challenges of everyday life. Social isolation makes the whole rehabilitation process particularly difficult [1]. Loneliness and social isolation affect quality of life and increase the likelihood of higher morbidity and earlier mortality [2]. Recent studies have shown that patients who have suffered a head injury are more prone to loneliness and depression and have a lower quality of life and emotional stability compared with healthy individuals. An important factor in this is the subjective aspect of isolation, which is considered as loneliness [3]. An inverse relationship has been found between the size of a social network and loneliness. Neuroticism also serves as an important mediating variable in the relationship between the size of the social network and the self-perception of loneliness [4].

It is important to recognize the perceived risk of loneliness, which affects not only those with multiple illnesses and conditions but also the elderly, as this awareness allows for appropriate interventions. One of the most basic interventions involves promoting a sense of community, which refers to a feeling of belonging and attachment to a specific community (residential, workplace, school, etc.). The severity of loneliness symptoms and the emotional support received are significant independent predictors of life satisfaction [5].

Integration or reintegration is defined as the activity of participating in a broad spectrum of community involvement, covering three main areas: independence, social and leisure activities and work or other productive activities [6]. The current practice of reintegrating patients after traumatic brain injury into their home environment in Slovenia includes psychological and social support during inpatient rehabilitation. A smaller percentage of patients join employment centers or brain injury patient associations after discharge. Neuropsychological care has proven to be very important, as neuropsychological deviations have a significant impact on performing activities of daily living and reintegration into the home environment [7].

In order to find out what problems patients with traumatic brain injury face after completing rehabilitation at our institute and what the objective level of their problems is, we used the Community Integration Questionnaire-Revised (CIQ-R) [8]. At the same time, the validity of the Slovenian translation of the questionnaire was checked. The assessment of the patient’s condition can help to prepare the patient and their relatives upon discharge for the problems that may arise in a certain period or over the years. In addition, we determined if (and if, where) there is a deficit in the follow-up and management of these patients.

## 2. Methods

### 2.1. Questionnaire

The Community Integration Questionnaire (CIQ) was developed in 1994 by Professor Barry Willer [9]. The questionnaire was originally intended for patients after traumatic head injury. In recent years, it has been translated into many languages [10,11,12,13,14,15,16], validated [7,17,18,19] and applied—at least for a rough evaluation or in an adapted version—to patients with other pathologies, such as post-stroke patients with aphasia [20], post-spinal cord injury [21,22,23,24], burns [25,26], brain tumors [27], muscular dystrophies, multiple sclerosis [12], amputations, developmental disorders and mental illness, and in the elderly [28].

The revised version (Community Integration Questionnaire-Revised, CIQ-R) from 2014 [7] provides additional information regarding the use of mobile devices and social networks. The additional Electronic Social Networking Subscale includes questions on the frequency of use of social networks and mobile devices for social networking. The questionnaire consists of 18 questions divided into four subscales: Home Integration (questions 1–6; example: Who usually prepares meals in your household?), Social Integration (questions 7–11; example: Approximately how many times a month do you usually visit friends or relatives?, Productivity (questions 12–15; example: Please check the answer that best corresponds to your current (during the past month) work situation.) and Electronic Social Networking (questions 16–18; example: How often do you make social contact with people by talking or text messaging using your phone?). The maximum number of points is 35, with more points representing better integration of the patient into the environment. The questionnaire takes 10 to 15 min to complete. It can be completed by a healthcare professional in a live conversation with the patient, over the phone, or by the patient independently [7].

We obtained permission to use the CIQ-R from Prof. Willer, who is the author of the original questionnaire and co-author of the revised version, in an email correspondence (31 March 2021). In the present study, we used the telephone completion of the questionnaire to make it easier for patients to understand and interpret the questions. The basic CIQ has already been translated into Slovenian [29], so we adapted the translation and additionally translated the extension. We then performed back-translation to harmonize the final version with the original English version.

### 2.2. Procedure

We searched our hospital’s database for patients with traumatic brain injury who were treated in our hospital in 2019 and 2020, and for whom no more than five years had passed since the injury (i.e., they were injured from 2016 onwards). We selected the patients who had at least one diagnosis of S06 (Laesio traumatica intracranialis) according to the International Classification of Diseases. Before the start of this study, we obtained their home addresses from their medical records and sent them a letter explaining the purpose of it and a consent form on which they entered their telephone numbers. If the patient was unable to sign or talk, we asked their legal representative to do so. Because of a low response rate after the first mailing, we sent a second invitation to participate in our study a month later.

Patients were telephoned twice, 14 days apart. During the call, they were assessed with the CIQ-R and the Functional Independence Measure (FIM) [29,30]. The FIM scores at admission to rehabilitation and discharge from our hospital were retrieved from our database. The patients were also asked about basic socio-demographic characteristics and internet access.

### 2.3. Data Analysis and Ethical Approval

The data were analyzed using IBM SPSS Statistics 27 (IBM Corp., Armonk, NY, USA). The internal consistency reliability of the CIQ-R and its subscales was assessed using Cronbach’s alpha coefficient and Guttman’s lambda-2 coefficient (with the RELIABILITY/MODEL = ALPHA and /MODEL = GUTTMAN commands, respectively). The reliability of the CIQ-R and subscale scores in terms of repeated measures, i.e., stability over time, was assessed using the intraclass correlation coefficient (ICC, with the RELIABILITY/ICC = MODEL (RANDOM) TYPE (CONSISTENCY) command). The correlation between the FIM and CIQ-R scores was assessed using Pearson’s *r*.

This study was based on a questionnaire, so no complications that could affect the patient’s health or well-being were expected. This research was approved by the Medical Ethics Committee of our institute.

## 3. Results

In 2020, there were 36 patients with traumatic brain injury treated at our institute. Overall, 25 of them were included in this study, as in four cases, more than five years had passed since the injury, and in one case, the cause of the injury was elsewhere. We also excluded five patients because they were over 65 years of age, and the address of one patient was unknown. The injuries occurred from 29 March 2017 to 17 August 2020; one occurred in 2017, two in 2018, seven in 2019 and 15 in 2020.

In 2019, 31 patients were treated, of whom 17 were included in this study because in five cases, more than five years had passed since the injury, one patient was from abroad, and we did not obtain their home address, six were over 65 years old and two patients had died. The injuries occurred from 27 January 2017 to 25 September 2019; two occurred in 2017, 10 in 2018 and five in 2019. In total, 42 of the 67 selected patients met the inclusion criteria. The year of the injury was 2017 in three cases, 2018 in 12 cases, 2019 in 12 cases and 2020 in 15 cases.

The patients suffered moderate to severe brain injuries (based on the Glasgow Coma Scale upon the arrival of paramedics), with two of the participants initially having mild injuries, but their condition later deteriorated.

Fifteen patients were willing to participate in this study (36% response rate). Only two women (13%) participated in this research, as the number of women was also lower among all participants (*n* = 6; 14%). Only one of the included patients (7%) had more than a high school education. Everyone had access to the internet (one with a borrowed device, the others with their own). About half of the participants lived in the city (*n* = 8, 53%), and half outside the city (*n* = 7, 47%).

The internal consistency of the overall score for the whole questionnaire was high (all estimates were above 0.8), but for the subscales, it was expectedly lower (ranging from 0.4 to 0.8) because of a smaller number of items (Table 1). This was most pronounced in the Productivity subscale.

**Figure 1:**
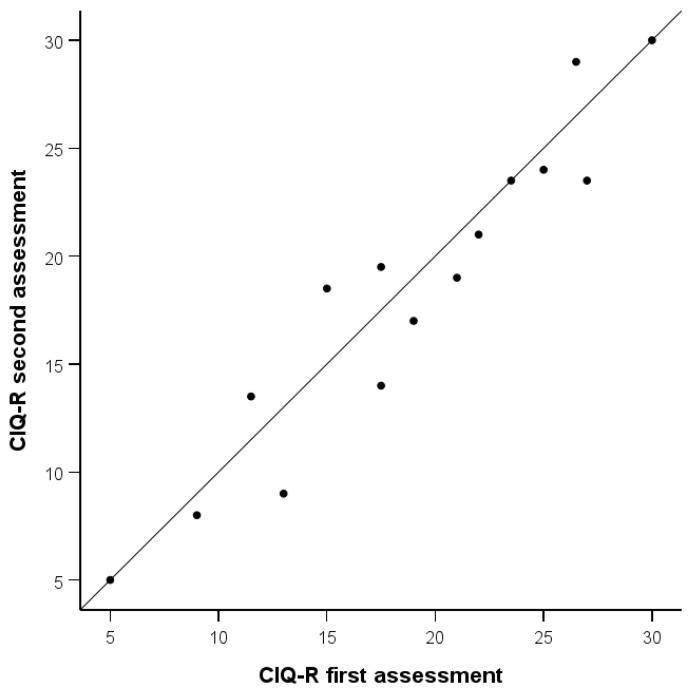
Total CIQ-R score upon first and second assessments (points on the diagonal indicate the same score upon both assessments).

When assessing the reliability of the questionnaire in terms of reassessment (Table 2, Figure 1), we observed very high stability of the total score (ICC = 0.95) and the subscale scores over the two weeks. For the Social Integration subscale, it was slightly lower but still high (ICC = 0.71).

**Figure 2:**
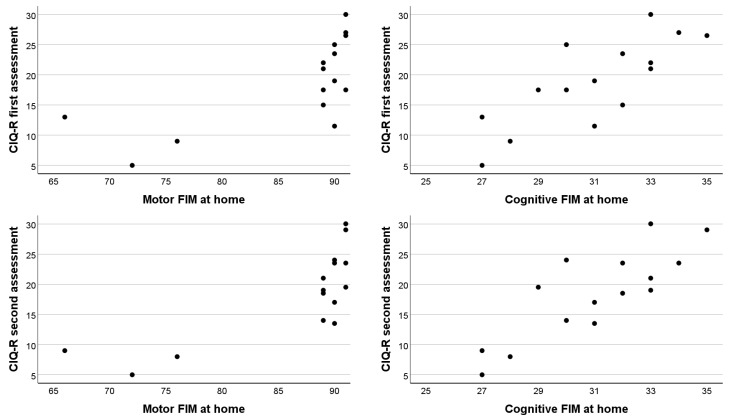
Scatter plots of the association between FIM subscale and total CIQ-R scores.

The comparison between the FIM and CIQ-R scores showed that only the FIM scores obtained at the time of the patients’ calls were statistically significantly associated with CIQ-R score (Table 3, FIM at home) and not FIM scores at the admission or discharge from the rehabilitation facility. The cognitive part of the FIM at home was clearly linearly related to the CIQ-R score (*r* = 0.78 and *r* = 0.83 with CIQ-R score obtained during the first and second assessments, respectively). The apparent association with the motor FIM was a consequence of the fact that three participants with low motor FIM scores (below 80) had very low overall CIQ-R scores (below 15 and below 10 during the first and second assessments, respectively), whereas the other participants had (almost) maximal motor FIM scores (89 to 91) while their CIQ-R scores varied widely (from 12 or 13 to 30 points; Figure 2).

Only one participant (7%) was involved in an association for patients after brain injury. According to the additional questions we asked, the majority of the participants were satisfied with their integration into the environment (*n* = 12, 80%), but 40% (*n* = 6) reported the integration being worse than in the pre-injury period.

## 4. Discussion

The questionnaire was successfully translated, and we demonstrated its potential suitability for clinical use. The estimated internal consistency reliability was high, and the stability of the total score as well as the subscale scores over the two weeks was very high.

We hypothesized that a lower FIM score at discharge from the rehabilitation facility would also imply a lower CIQ-R later and that the current FIM and CIQ-R scores would be positively correlated, i.e., that a lower FIM would coincide with a lower CIQ-R. We did not find a notable correlation between the FIM score during the rehabilitation (either at admission or at discharge) and subsequent integration into the home environment. A statistically significant association was observed between the CIQ-R and the FIM at home, whereby a linear association was observed only with the cognitive part of the FIM, while the motor part was correlated with CIQ-R only for those with very low scores.

A limiting factor in our study was the poor willingness of eligible patients to participate, as only 15 (36%) of the 42 included patients were willing to participate. There is a possibility that only the better-involved patients were willing to participate. This can be interpreted from the perspective that poorly integrated patients are influenced by anxiety and depression and are, therefore, mentally and physically unprepared to participate in research or, in our case, a telephone interview. The relatively low response rate may also be the result of the fact that the patients were asked to give us their telephone number and to be interviewed twice within two weeks. Additionally, despite careful and precise explanations, there is a possibility that some patients did not understand the purpose of this research and, therefore, chose not to participate. Hence, the possibly excluded patients with a less favorable rehabilitation outcome might have been those with lower cognitive abilities. A more general limitation of this study is that it focused on social aspects without considering falls [31], which are also an important issue for inpatient rehabilitation and subsequent integration of patients after head injuries.

The literature states that the severity of a head injury, particularly in terms of the duration of amnesia, affects the outcome of successful integration into the home environment [10,32,33]. Patients who were active in social networks achieved better integration, especially in social connections [10]. Being included in the environment has been associated with a good quality of life, satisfaction and less emotional distress [34]. Like previous studies, we found that most patients after head injuries are less integrated into their environments [35]. A possible solution for better integration is to introduce a program with a navigator, i.e., a person who takes care of patients after discharge, is available to them in case of any problems and is a link between the patient and the doctor and also between patients of the same pathology. It is reported that this can improve integration, independence and functional abilities and reduce hospital readmissions and falls [36]. Strategies to facilitate the integration include cognitive, physical and emotional rehabilitation, as well as facilitating a strong support system in the home environment and community [33].

It should be considered that this study was conducted during the coronavirus pandemic, which certainly had an impact on patient activity and socialization. The epidemic has had an impact on all people in terms of socializing and probably even more so on our patients, who were expected to be less integrated into their environments even before the epidemic.

In practice, the CIQ-R could be used in follow-up examinations, where, in addition to the clinical and functional status, we could obtain better insight into the patient’s involvement. In the case of poor involvement, we could advise on activities to improve socialization, perhaps by joining patient associations. This would encourage patients to live active lives and feel useful. Consequently, we can prevent the development of anxiety and depression. In addition, before discharge, patients and relatives could be prepared for the difficulties they may face in the home environment by providing them with information on who to contact in the case of problems with integration. We could also introduce a post-discharge navigator [36] or offer support via telehealth [37]. It might also be useful to distribute leaflets to join the patient associations and to actively involve the associations with rehabilitation facilities. In the case of expecting problems with integration, the hospital’s social services should be involved.

Future research using the Slovenian translation of the CIQ-R should include a larger sample, assess the relationship with cognitive status and test the applicability to other pathologies.

## 5. Conclusions

A successful integration of patients into their home and work environments is the ultimate goal of rehabilitation. Our research has confirmed that our patients have difficulties integrating back into their home environments.

The Community Integration Questionnaire-Revised (CIQ-R) was successfully translated into Slovenian and is available for use in clinical practice. A follow-up examination after completing rehabilitation, including assessment with the CIQ-R, provides insight into how the patient functions in the home environment, how much he/she is involved in social activities and the location of the deficits.

It was surprising to find out that very few patients participate in the associations for patients after traumatic brain injury. Joining associations could help them to integrate better into their home and work environments and could provide support in terms of conversations, sharing experiences and exchanging advice to help them cope with their injuries. In order to facilitate integration into the environment, it is essential to establish a network of patients. Good patient integration is important and should not be overlooked when preparing for a patient’s discharge from a rehabilitation facility.

## Figures and Tables

**Table 1:** Estimates of internal consistency reliability of the Community Integration Questionnaire-Revised (CIQ-R).

	First Assessment		Second Assessment	
Scale	Alpha	Lambda-2	Alpha	Lambda-2
CIQ-R	0.81	0.84	0.82	0.85
Home Integration	0.70	0.78	0.77	0.82
Social Integration	0.52	0.57	0.48	0.50
Productivity	0.38	0.38	0.45	0.45
Electronic Social Networking	0.55	0.60	0.62	0.63

**Table 2:** Estimates of test–retest reliability of the Community Integration Questionnaire-Revised (CIQ-R).

Scale	ICC	(95% CI)
CIQ-R	0.95	(0.86, 0.98)
Home Integration	0.84	(0.59, 0.94)
Social Integration	0.71	(0.34, 0.89)
Productivity	0.99	(0.98, 1.00)
Electronic Social Networking	0.96	(0.87, 0.99)

Legend: CI—confidence interval.

**Table 3:** Association between FIM and CIQ-R scores.

		First Assessment		Second Assessment	
Time-Point	FIM	*r* with CIQ-R	*p*	*r* with CIQ-R	*p*
Admission	Motor	0.13	0.638	0.16	0.581
	Cognitive	0.30	0.282	0.33	0.234
	Total	0.20	0.470	0.23	0.409
Discharge	Motor	0.25	0.377	0.24	0.397
	Cognitive	0.37	0.180	0.41	0.134
	Total	0.32	0.240	0.33	0.231
At home	Motor	0.69	0.005	0.77	0.001
	Cognitive	0.78	0.001	0.83	<0.001
	Total	0.74	0.002	0.82	<0.001

## Data Availability

The original contributions presented in this study are included in this article. Further inquiries can be directed to the corresponding author.
